# Neurodevelopmental Disorders: Effect of High-Fat Diet on Synaptic Plasticity and Mitochondrial Functions

**DOI:** 10.3390/brainsci10110805

**Published:** 2020-10-31

**Authors:** Eduardo Penna, Amelia Pizzella, Fabiano Cimmino, Giovanna Trinchese, Gina Cavaliere, Angela Catapano, Ivana Allocca, Jong Tai Chun, Angelo Campanozzi, Giovanni Messina, Francesco Precenzano, Valentina Lanzara, Antonietta Messina, Vincenzo Monda, Marcellino Monda, Carla Perrone-Capano, Maria Pina Mollica, Marianna Crispino

**Affiliations:** 1 Department of Biology, University of Naples Federico II, 80126 Naples, Italy; eduardo.penna@unina.it (E.P.); amelia.pizzella94@hotmail.it (A.P.); fabiano.cimmino@unina.it (F.C.); giovanna.trinchese@unina.it (G.T.); gina.cavaliere@unina.it (G.C.); angelacatapano@me.com (A.C.); iv.allocca@gmail.com (I.A.); crispino@unina.it (M.C.); 2 Department of Pharmacy, University of Naples Federico II, 80131 Naples, Italy; perrone@unina.it; 3 Department of Biology and Evolution of Marine Organisms, Stazione Zoologica Anton Dohrn, 80121 Naples, Italy; chun@szn.it; 4 Department of Medical and Surgical Sciences, University of Foggia, 71122 Foggia, Italy; angelo.campanozzi@unifg.it; 5 Department of Clinical and Experimental Medicine, University of Foggia, 71122 Foggia, Italy; giovanni.messina@unifg.it; 6 Department of Mental Health, Physical and Preventive Medicine, Clinic of Child and Adolescent Neuropsychiatry, Università degli Studi della Campania “Luigi Vanvitelli”, 80138 Naples, Italy; francesco.precenzano@unicampania.it (F.P.); valentina.lanzara@unicampania.it (V.L.); 7 Department of Experimental Medicine, Section of Human Physiology and Unit of Dietetics and Sports Medicine, Università degli Studi della Campania “Luigi Vanvitelli”, 80138 Naples, Italy; antonietta.messina@unicampania.it (A.M.); marcellino.monda@unicampania.it (M.M.); 8 Department of Experimental Medicine, Università degli Studi della Campania “Luigi Vanvitelli”, 81100 Caserta, Italy; mondavincenzo@gmail.com; 9 Institute of Genetics and Biophysics “Adriano Buzzati Traverso”, CNR, 80131 Naples, Italy

**Keywords:** neurodevelopmental disorders, high-fat diet, synaptic plasticity, mitochondria, synaptic protein synthesis

## Abstract

Neurodevelopmental disorders (NDDs) include diverse neuropathologies characterized by abnormal brain development leading to impaired cognition, communication and social skills. A common feature of NDDs is defective synaptic plasticity, but the underlying molecular mechanisms are only partially known. Several studies have indicated that people’s lifestyles such as diet pattern and physical exercise have significant influence on synaptic plasticity of the brain. Indeed, it has been reported that a high-fat diet (HFD, with 30–50% fat content), which leads to systemic low-grade inflammation, has also a detrimental effect on synaptic efficiency. Interestingly, metabolic alterations associated with obesity in pregnant woman may represent a risk factor for NDDs in the offspring. In this review, we have discussed the potential molecular mechanisms linking the HFD-induced metabolic dysfunctions to altered synaptic plasticity underlying NDDs, with a special emphasis on the roles played by synaptic protein synthesis and mitochondrial functions.

## 1. Introduction

Neurodevelopmental disorders (NDDs) comprise a heterogeneous group of neuropathologies. These disorders usually appear during infancy or childhood, and depend on the aberrant development of the nervous system, which leads to impairments of various neural functions such as cognition, communication, social behavior and motor skills. According to the *Diagnostic and Statistical Manual of Mental Disorders, version 5*, from the American Psychiatric Association, NDDs include autism spectrum disorder (ASD), attention-deficit/hyperactivity disorder (ADHD), intellectual disabilities, specific learning disorder, communication disorders and motor disorders [1]. Nonetheless, the diagnosis of NDDs is complicated by the fact that many clinical symptoms are not unique to a single NDD, but there is a great deal of overlap among different NDDs [1,2]. In addition, the onset of the disease and its detection are two different things. For some NDDs such as Rett’s syndrome (RTT), whose genetic causality is clearly established, it is conceivable that the disease might have been on its way during the early stage of development. However, the diagnosis is typically made between 6 and 18 months when the behavioral symptoms are becoming evident. Therefore, it is difficult to establish the precise onset of NDDs, although its progress might be attributable to impaired synaptic maturation and neuronal connectivity [3]. Indeed, several data have indicated that a common feature of NDDs is the alteration of the molecular pathways underlying synaptic plasticity [4,5]. Synaptic plasticity is the fascinating capacity of the brain to respond to stimuli by changing the synaptic strength and modifying neuronal circuits. This activity-dependent modification of the synaptic efficacy and cytoarchitecture of neuronal wiring is responsible for the ability of the brain to integrate temporary experiences into consolidated behavior, affecting thoughts, feeling and memory. Thus, analyzing the molecular mechanisms behind impaired synaptic plasticity will greatly help us comprehend the cellular and physiological basis of NDDs.

It is noteworthy that children with developmental anomalies may also display a variety of non-specific functional impairments in addition to improper brain maturation. These non-specific impairments often make the child more susceptible to other sources of disabilities such as infection, trauma and altered eating habits [2]. In recent years, it has been emphasized that appropriate dietary behavior is highly relevant to maintenance of a state of wellness. It is well-known that a chronic high-fat diet (HFD, with 30–50% fat content) intake is one of the key factors contributing to the development of obesity. Indeed, obesity may be now viewed as a multifactorial pathology, which is caused by complex interactions among several elements, including not only HFD but also various behavioral, genetic, epigenetic, socioeconomic and cultural factors. Essentially, obesity results from a long-term energy imbalance where dietary intake far exceeds energy expenditure. The incidence of obesity has significantly increased in recent decades, presumably due to the more sedentary lifestyle, and to the easier access to calorie-dense food [6]. Adding fat to commercial food improves its palatability and makes it cheaper and more energy-dense. These are the reasons why fatty foods are preferentially selected by consumers [7]. HFD-induced obesity often leads to low-grade chronic inflammation with several pathological consequences such as increased risk of diabetes, cardiovascular disease and hypertension [8,9,10,11]. As a consequence of HFD, the adipose tissue increases due to the hypertrophy and hyperplasia of adipocytes. The hypertrophy is a potential stress factor for the endoplasmic reticulum. This may cause inflammation and leads to insulin resistance in adipocytes [9]. Fat cells going towards lipolysis increase circulating levels of free fatty acids. That in turn elevates the levels of proinflammatory cytokines [12,13,14]. The resulting metabolic inflammation has an impact not only on fat cells, but on the whole body, including the central nervous system (CNS). As the brain is one of the most active organs in the human body, it is highly influenced by nutritional elements. Indeed, HFD has been shown to be tightly associated with neuropathologies [15,16,17,18]. For instance, some data reported a bidirectional association between obesity and depression [19,20,21], suggesting that obesity can directly affect mood. Conversely, stress-related mental disorders can lead to changes in diet habits affecting body weight [22]. Thus, it appears that diet, especially HFD, has a considerable effect on the physical and mental health of people. In this review, we have examined the mechanisms underlying the HFD-induced pathological processes, including their potential influence on NDDs.

## 2. Effects of HFD during Pregnancy and in Childhood

Several lines of studies have demonstrated that maternal obesity during pregnancy has extensive negative effects on the offspring’s brain functions. In particular, in the rodent animal model, it has been shown that maternal HFD negatively influences not only the development of the fetal hippocampus [23], but also the offspring’s cognitive functions later on. This may be due to impaired neurogenesis and the alterations in dendritic arborization, hippocampal lipid peroxidation and brain-derived neurotrophic factor (BDNF) expression [24,25,26,27,28]. Therefore, it is not surprising that maternal HFD is linked to higher incidence of NDDs in the offspring [29,30,31,32]. Interestingly, in mouse, the negative effect of maternal HFD on the offspring’s brain development was shown to depend on altered expression of epigenetic regulators [33]. A recent comprehensive epidemiological study supported the link between maternal diabetes and the increased risk of ASD and ADHD in children, although the effect sizes were moderate. It is still unclear if the link is due to obesity-associated intrauterine mechanisms or to maternal genetic factors [34]. Using animal models, it has been suggested that some of the molecular mechanisms underlying the increased risk of NDDs in the offspring of obese mothers involve: (i) increased release of placental proinflammatory cytokines [35], (ii) altered release of metabolic hormones such as insulin and leptin [36,37], (iii) abnormalities in serotonergic and dopaminergic systems in offspring [38,39]. For the latter point, it is noteworthy that serotonin (5-hydroxytryptamine, 5-HT) exerts morphogenetic actions on the brain, influencing several neurodevelopmental processes during embryonic and early postnatal life [40,41]. Interestingly, decreased levels of 5-HT were reported in the hippocampus of mice fed with HFD, which also exhibited anxiogenic and depressive-like symptoms [42]. The numerous roles played by 5-HT in the brain depend on its interaction with different subtypes of receptors, which are widely distributed in the brain and grouped in seven distinct classes (from 5-HT1R to 5-HT7R). Among them, 5-HT7R plays a crucial role in modulation of neuronal morphology and synaptic plasticity, contributing to the establishment of brain connectivity during development and to remodeling of the neuronal wiring in the mature brain [40,41,43,44,45]. Thus, the activation of 5-HT7R has been suggested as a therapeutic approach for NDDs which are tightly associated with altered synaptic plasticity [46].

Childhood and adolescence represent two crucial periods in brain maturation, which are particularly sensitive to the deleterious neurocognitive effects of HFD [47,48]. Consumption of HFD in juvenile mice often decreases hippocampal neurogenesis, affects synaptic functions and impairs memory and cognitive abilities [48,49]. Thus, it is conceivable that HFD during childhood may contribute to worsen the symptoms of NDDs. In this regard, appropriate management of diet could serve as an alternative therapeutic approach for NDDs.

## 3. HFD and Neuroinflammation

Neuroinflammation is generally considered as activation of the neuroimmune glial cells, e.g., microglia and astrocytes, into proinflammatory states [50,51]. Mild activation of these cells usually displays neuroprotective effects, while strong and persistent activation causes cytokine overexpression and the release of reactive oxygen species (ROS), the latter of which leads to neuronal damages and synaptic dysfunction [52]. Interestingly, the damages due to inflammation and oxidative stress in multiple organs, including the CNS, are found both in obesity and during the aging processes [53]. Some of the pathological consequences of obesity, i.e., insulin and leptin resistance, mitochondrial impairment and oxidative stress, may anticipate the aging process that is linked to higher susceptibility to neurodegenerative diseases [54,55,56,57,58]. In particular, the age at which HFD starts and its duration in life are important factors in determining obesity and its pathological prognosis. Thus, the interaction between age and HFD potentiates their negative effects on metabolic parameters [58].

It is noteworthy that one of the common features of neuroinflammation and neuropathologies is altered synaptic plasticity [59,60]. Since it has been widely demonstrated that diet influences synaptic plasticity, and that HFD consumption is linked to NDDs [29], it is intriguing to see which synaptic mechanisms are affected by HFD to unveil some of the molecular pathways underlying NDDs.

## 4. Local Protein Synthesis: A Molecular Mechanism Underlying Synaptic Plasticity

The impairment of synaptic plasticity contributes to the pathophysiology of several disorders of the nervous system [61]. Thus, the molecular mechanisms of synaptic plasticity underlying continuous remodeling of pre- and postsynaptic subcellular zones are crucial to the physiology and pathology of the nervous system.

In this context, an emerging idea is that an intriguing role is played by local protein synthesis in the synapse [62,63,64]. For a long time, it was generally believed that the soma was the exclusive site of protein synthesis in the neuron, and that the axonal and presynaptic proteins were delivered to their destination exclusively by axoplasmic transport. A neuron is a highly polarized cell typically endowed with several dendrites and one single axon arising from the cell body. The axon, in some neurons, may be hundreds of centimeters long or extensively branched at its edge. Each presynaptic terminal establishes a contact with other cells, leading to the formation of a complex neuronal network that allows the nervous system to carry out its physiological functions. Due to this unique morphology, the synaptic terminals can be up to a meter away from the cell body, and the molecular composition of each synapse in the same axon may be distinct and differentially regulated [65]. Thus, the possibility that mRNA translation takes place on demand and locally within the presynaptic area may greatly contribute to the ability of the synaptic regions to rapidly respond to the changes of local environment. Acceptance of this idea took a long time, but it is now well-established that several different proteins are locally synthetized in the axons and nerve terminals in different model systems. To name a few, among locally synthesized proteins are synaptosomal-associated protein, 25kDa (SNAP-25) [66] and Munc 18-1 [67], ribosomal proteins [68], cytoskeleton components such as neurofilament proteins [69], the amyloid precursor protein (APP) [70] and a protease inhibitor cystatin B, the latter of which has been recently demonstrated to have a key role in synaptic plasticity [71,72,73].

The synaptic machinery of protein synthesis plays a crucial role during development, supporting axonal outgrowth, branching and synaptogenesis. It is also active during adulthood in regulating synaptic transmission and axonal survival [74,75]. Several sets of data have demonstrated that the synaptic system of protein synthesis makes a significant contribution to synaptic plasticity. For instance, in mature mammalian brains, synaptic translation is involved in neurotransmitter release during long-term plasticity [76] and supports maintenance of the visual circuits [75]. In addition, modulation of synaptic protein synthesis by learning was demonstrated both in young adult and old rats, although the correlations between the newly synthesized proteins and the behavioral responses were subtly different in the two age groups [77,78]. Thus, aging appears to affect the adaptability of the synaptic system of protein synthesis. Interestingly, it was also demonstrated that dysregulation of this local mechanism of protein synthesis contributes not only to neurodegenerative diseases [70,79], but also to neurodevelopmental disorders such as Fragile X syndrome [80,81,82]. Therefore, to find a way to correct the altered system of protein synthesis located in the synaptic area has great relevance from a translational neuroscience point of view, offering new hope for treating neurodevelopmental disorders.

## 5. Effects of HFD on Synaptic Plasticity

Several findings have indicated that synaptic dysfunction could also occur in the brain as a result of diet-induced obesity. In particular, HFD causes neuroinflammation through overexpression of proinflammatory cytokines, inducible nitric oxide synthase (iNOS), tumour necrosis factor alpha (TNF-α), as well as through a significant increase of mTOR (mammalian target of rapamycin) activation, which is involved in the pathogenesis of both metabolic and neurological diseases [83,84,85]. Furthermore, HFD influences the activity of 5’ adenosine monophosphate-activated protein kinase (AMPK) [86], a cell’s energy sensor that is activated by the decrease of cellular energy status. In response to it, ATP production is increased by triggering catabolic pathways and inhibiting anabolic pathways [87]. Interestingly, the activation of AMPK regulates the expression of the immediate early genes (IEGs) via the protein kinase A (PKA)/cAMP response element-binding protein (CREB) pathway. IEGs expression is induced by synaptic activation, a highly energy-demanding process responsible for the formation of long-term memory [88]. Thus, AMPK could be one of the key molecules linking metabolic pathways to memory formation.

It is also known that HFD consumption may lead to altered insulin signaling [9]. For a long time, insulin was considered a peripheral hormone, unable to cross the blood–brain barrier and to affect the CNS. Instead, it is now well-demonstrated that pancreatic insulin is transported to the brain via cerebrospinal fluid, thereby bypassing the blood–brain barrier owing to dedicated carriers [89]. In this context, it should also be noted that insulin might be even synthesized in the brain, as shown by the data in cultured rat brain neurons that can produce and release insulin under depolarizing conditions [90]. This topic is still matter of debate [89,90,91,92]. Nevertheless, the importance of insulin signaling in the brain is demonstrated by the fact that insulin receptors are highly abundant throughout the CNS, particularly in selected regions such as the olfactory bulb, hypothalamus, cerebral cortex, cerebellum, hippocampus and striatum [89]. Interestingly, brain insulin regulates both systemic and cerebral glucose metabolism, as well as neurotransmission and synaptic plasticity. For instance, exogenous insulin administration activates the phosphatidylinositol 3-kinases (PI3K)/protein kinase B (AKT) pathway and promotes long-term potentiation (LTP) induction in hippocampal synapses. Since synaptic transmission and synaptic plasticity are correlated with learning and memory processes, it was concluded that insulin is able to actively modulate hippocampal-dependent cognitive events [93]. As a consequence, HFD-induced insulin resistance might be associated with memory and learning decline. Strikingly, insulin receptors are localized in the synaptic area, a finding strongly suggesting that insulin may play a signaling role in the synapse that is quite distinct from the conventional effect on systemic metabolism [94,95]. Elucidating this role at synapse will provide important insights into the synaptic function and plasticity. From this point of view, an intriguing hypothesis might be that insulin’s action on learning and memory depends on its modulation of synaptic protein synthesis [96]. Similar to insulin, it has been reported that leptin signaling is also involved in learning through the regulation of neurotransmitter release and synaptic plasticity [97,98,99]. In the hippocampus, it has been demonstrated that leptin signaling pathways facilitate synaptic transmission by selectively modulating N-Methyl-D-aspartate (NMDA) receptors [100]. As a consequence, leptin is able to convert short-term potentiation of synaptic transmission into LTP and thereby contributes to neuronal plasticity. The key role played by leptin in the brain has been demonstrated also by its neurotrophic effect on hypothalamic neurons during development [101]

In animal models, obesity-induced microglia activation and neuroinflammation are associated with impaired LTP, decreased synaptic density and dysregulated expression of the genes related to synaptic plasticity [102]. In line with that, it was also reported that HFD decreases the expression levels of several mRNAs related to neurogenesis and synaptic plasticity, as well as the levels of non-coding RNAs, indicating a complex control of diet on protein expression [103]. In particular, HFD affects the hippocampal expression levels of SNAP-25, a component of the soluble *N*-ethylmaleimide-sensitive factor attachment protein receptor (SNARE) protein complex that is directly involved in neurotransmitter release [104]. Interestingly, the intra-axonal synthesis of this protein is required during development for the correct formation of synapses and for the release of the synaptic vesicles from the presynaptic terminal [66]. Altogether, these data suggest that HFD may eventually alter the synaptic synthesis of SNAP-25, leading to impaired synaptic plasticity. Accordingly, in rodent models, it appears that HFD negatively affects synaptic plasticity and electrophysiological responses in the hippocampus, a brain region crucially involved in the management of information and emotions. Consistently, HFD also tends to induce multiple behavioral alterations mainly related to increased anxiety [105,106].

It has been shown that HFD-induced neuroinflammation and oxidative stress not only have an impact on synaptic functions but are also correlated with expression levels of BDNF [16,42,107,108,109]. BDNF is a neurotrophin implicated in several mechanisms of neuronal plasticity [110,111,112]. In particular, BDNF is essential for fine tuning of synaptic transmission and for LTP in the hippocampus. BDNF has both short-term and a long-term effects on synaptic plasticity, including the modulation of the synaptic system of protein synthesis [113]. Interestingly, a sharp decrease of BDNF expression was observed in the synaptic regions of HFD-treated mice, leading to the hypothesis that the reduced BDNF level may contribute to decreased synaptic protein synthesis [16]. It is noteworthy that synaptic protein synthesis is also crucial for local mitochondrial activity, and its alteration may lead to numerous diseases of the nervous system [114]. The interdependent interaction between the local translation machinery and mitochondria at the synapse may be a novel mechanism underlying synaptic plasticity [115]. In line with that, it should be noted that some mitochondrial proteins, such as the ones being involved in mitochondria biogenesis, are synthesized in synaptic regions and locally transferred into mitochondria [116].

The growing importance of dietary manipulation is reflected by the current trend of using it in a therapeutic approach to treat some neurological disorders. In particular, the ketogenic diet (KD) drew some attention for its anti-epileptogenic effects demonstrated both in animal models and in the epilepsy patients subjected to the therapy [117,118]. Containing high-fat and sufficient protein but very low carbohydrate, KD is designed to induce hepatic production of ketone bodies (e.g., acetoacetate and β-hydroxybutyrate) [117]. The low availability of carbohydrates forces the neurons to rely on ketone bodies for energy. Thus, it is no surprise that KD influences the brain functions. A growing body of evidence shows that KD can alleviate some core symptoms of ASD [119,120,121], and mitigate social and self-directed repetitive behavior in animal models for ASD [122,123,124]. Although these studies suggest KD as a promising therapeutic treatment for ASD, the molecular mechanisms underlying its beneficial effects need to be investigated further [125].

## 6. The Role of Mitochondria in NDDs

The brain requires a large amount of energy for its physiological need to maintain synaptic activity and connectivity. It is estimated that the brain consumes about 20% of oxygen and 20–25% of glucose that are available to the body [126]. In fact, synaptic excitability, ion influx, management of cytoskeletal dynamics and the membrane potential, as well as neurotransmitter release, all require hydrolysis of a large amount of ATP [127,128,129]. This enormous energy demand depends on mitochondria, which are the main generators for cellular energy. Accordingly, the energy provided by the brain mitochondria is higher than in other organs. Indeed, impaired mitochondria functions impact the brain much more than other organs [130]. In addition to the bioenergetics roles for the cells, mitochondria are also involved in the synthesis of molecules related to inflammation and to the production and consumption of ROS. Thus, mitochondrial dysfunction can be either the cause or the consequence of inflammatory processes and may trigger metabolic adaptations leading to protective or detrimental pathways [131]. The brain is particularly susceptible to oxidative stress mainly due to its (i) high lipid content that is prone peroxidation, (ii) high energy demand requiring elevated oxygen consumption and (iii) relatively low levels of antioxidants [132]. The interactions among oxidative stress, neuroinflammation and mitochondrial dysfunction are likely to create a synergistic loop that amplifies the deficits.

As aforementioned, mitochondria located in the synaptic area of a neuron play a variety of roles. It should be added that, besides providing ATP for synaptic functions, mitochondria also serve as a buffer for calcium ions and support the local system of protein synthesis that is necessary for synaptic plasticity [133,134]. Thus, their dysfunction is directly linked to both neuroinflammation and compromised synaptic plasticity. Indeed, it was recently reported that the negative influence of HFD consumption on brain cortex bioenergetics was more pronounced in the synaptic region due to the markedly impaired mitochondrial function in this area [16].

**Figure 1:**
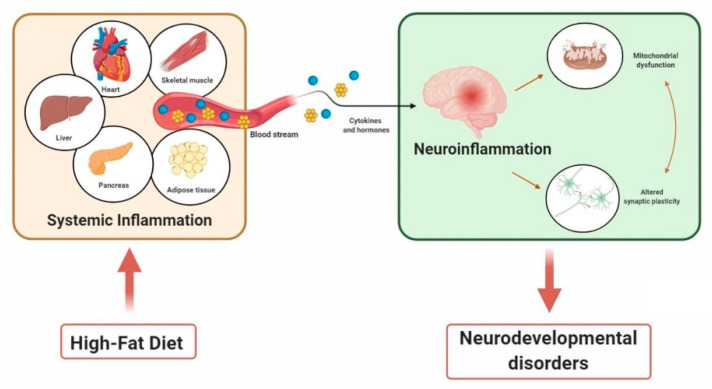
A general scheme of high-fat diet (HFD) being linked to inflammation and neurodevelopmental disorders (NDDs). HFD affects several organs, leading to systemic inflammation. Proinflammatory cytokines and hormones, which are released in response to the inflammation, induce neuroinflammation, accompanied by mitochondrial dysfunctions and altered synaptic plasticity in the brain, thus contributing to NDDs.

The HFD-related mitochondrial dysfunctions in synaptic areas imply their involvement in NDDs that are characterized by impaired synaptic plasticity. Accordingly, in both animal models and human patients, one of the major features of ASD is altered mitochondrial functions [113,114]. The mitochondrial involvement in ASD was indirectly indicated for the first time in 1985 when it was reported that a subgroup of ASD children displayed lactic acidosis [135]. The significant link between mitochondria and ASD was confirmed several years later, and it is now well-accepted that numerous children with ASD have mitochondrial dysfunctions, including biochemical and genetic abnormalities of these organelles [136]. In line with that, in postmortem brains of autistic patients, it was found that expression levels of several genes related to mitochondrial electron transport complex were significantly reduced [137]. Recently, it was reported that hippocampal mitochondria of an ASD mouse model exhibited a reduced respiratory capacity, leading to increased oxidative stress [138]. These results parallel with the ones obtained in a mouse model of RTT, where the mitochondrial respiratory chain was impaired and free radicals were overproduced [139]. Structural and functional alterations of mitochondria were repeatedly reported in both RTT patients and animal models. It has been postulated that the impaired mitochondrial functions and the consequent elevated levels of oxidative stress depend on the absence of MeCP2, the RTT causal gene [140]. Several recent data indicated alteration of mitochondrial dynamics as a key factor in the pathogenesis of NDDs, although the underlying mechanisms are still unclear [141] (Figure 1).

## 7. Conclusions

NDDs are a group of distinct diseases displaying a wide spectrum of symptoms severity, which can be devastating in some cases. For many of them, there is no cure so far, although some treatments to relieve the symptoms have been identified. Here we have reported some recent data indicating that a common feature of NDDs is neuroinflammation and altered synaptic plasticity, which are possibly linked to impaired local protein synthesis and mitochondrial functions in synaptic areas. Such deregulation may play an important role in the pathogenesis of these diseases. Thus, it is worth investigating further the potential molecular pathways. Based on our discussion, it is possible to hypothesize that a suitable diet, aiming at improving the mitochondrial dysfunction in synapse, can be an innovative preventive or therapeutic approach to treat NDDs.
